# Integrated problem-based learning versus lectures: a path analysis modelling of the relationships between educational context and learning approaches

**DOI:** 10.1080/10872981.2018.1489690

**Published:** 2018-07-03

**Authors:** Marie-Paule Gustin, Milena Abbiati, Raphael Bonvin, Margaret W. Gerbase, Anne Baroffio

**Affiliations:** a Department of public health, Biostatistics, Institute of pharmaceutic and biological sciences, Lyon, France; b Emerging pathogens laboratory - Fondation Mérieux, International Center for Infectiology Research(CIRI), Lyon, France; c Unit of Development and Research in Medical Education, University of Geneva Faculty of Medicine, Geneva, Switzerland; d Medical Education Unit, Faculty of science and medicine, University of Lausanne, Lausanne, Switzerland

**Keywords:** Educational context, integrated curriculum, learning approaches, medical education, path analysis models, problem-based learning, student perception of the educational context, undergraduate medical students

## Abstract

Students’ approaches to learning are central to the process of learning. Previous research has revealed that influencing students’ approaches towards deep learning is a complex process and seems much more difficult than expected, even in student-activating learning environments. There is evidence that learning approaches are impacted not only by the learning environment, but also by how students perceive it. However the nature of the links between the environment itself, the way in which it is perceived by students and students’ learning approaches is poorly understood. This study aimed at investigating the relationships between students’ perception of their educational context and learning approaches in three learning environments differing by their teaching formats (lecture or problem-based-learning PBL) and integration level of the curriculum (traditional or integrated). We tested the hypothesis that a PBL format and an integrated curriculum are associated to deeper approaches to learning and that this is mediated by student perception. The study sample was constituted of 1394 medical students trained respectively in a traditional lecture-based (*n* = 295), in an integrated lecture-based (*n* = 612) and in an integrated PBL-based (*n* = 487) curricula. They completed a survey including the Dundee-Ready-Educational-Environment-Measure (students’ perceptions of the educational environment) and the Revised-Study-Process-Questionnaire (learning approaches). Data were analysed by path analysis. The model showed that the learning environment was related to students’ learning approaches by two paths, one direct and one mediated via students’ perception of their educational context. In the lecture-based curricula students’ used deeper approaches when it was integrated and both paths were cumulative. In the PBL-based curriculum students’ did not use deeper approaches than with lectures, due to opposite effects of both paths. This study suggested that an integrated lecture-based curriculum was as effective as a PBL curriculum in promoting students’ deep learning approaches, reinforcing the importance of integrating the curriculum before choosing the teaching format.

## Introduction

Learning approaches are central to students’ learning. The concept of learning approaches was initially proposed by Marton and Säljö [1] and later extended [2–4]. These authors defined that students had different intentions when starting a learning task and used different learning processes and strategies to deal with it. Students were defined as ‘deep learners’ if they tried to understand the meaning of what they were learning, related information to prior knowledge, looked for underlying principles and critically evaluated their knowledge and also the conclusions they draw. These students were driven by an intrinsic interest. Conversely, if students were reproducing content, memorizing and rote learning in order to pass the tests, they were defined as ‘surface learners’. These students were driven by a fear of failure. Students’ use of deep approaches were considered desirable since they were impacting students’ learning outcomes [5]: several authors showed that students’ use of deep approaches predicted their academic performance, and that students who passed their exams very well had used deeper approaches than those who had done less well [6–8].

In 1993, J. Biggs [5] proposed a model of the learning process, which postulated that learning approaches were influenced on the one hand by the characteristics of the students and on the other hand by their learning environment or educational context (‘educational context’ will be used in this paper). Thereafter, much effort has been given on fostering deep approaches to learning in order to promote deep and meaningful learning in particular through student-centred teaching methods [9]. More recently, approaches to learning were shown not to be a stable psychological trait [9] knowing that students can adopt one approach or the other depending on the educational context in which they are learning [4,10]. However, fostering a deep approach has revealed quite difficult [11,12]. In 2010, Baeten and colleagues reviewing the studies addressing the effects of educational context on students’ approaches to learning, confirmed that ‘influencing students’ approaches towards deep learning is a complex process’ and that little is known about how factors interact and relate one to the other [13]. In addition, this review highlighted an issue raised earlier namely that it was students’ perceptions of their educational context, more than the context in itself, which influenced how a student learned [13,14]. Thus, in addition to contextual factors such as student interactivity, group work, or assessment type, students’ perception of workload, teaching quality, or of relevance to professional practice, were some of the factors impacting students’ deep learning. The educational context such as it is perceived by students, called ‘climate’ by Genn [15], has been described as the ‘soul and spirit of the medical school’ influencing students’ behaviour. In particular, it is related to students’ satisfaction and motivation [16], but most importantly to their achievement and academic performance [17,18].

The perception of the educational context can change when curricula are changed [19]. The operationalization of the curriculum generates an educational context [15] that should ideally promote an autonomous, self-motivated, independent, problem-solving learner. Since the early 1990s, a major trend in medical curricula around the world has been about integrating the teaching of basic, clinical and psychosocial sciences [20]. The principle is to construct multidisciplinary system-based blocks integrating discipline-based threads [21,22]. By breaking down the barriers between basic and clinical sciences, integrated curricula are thought to promote knowledge retention and development of clinical skills [20]. They also aim at providing an opportunity for students to acquire a more relevant and less fragmented knowledge. In addition, influenced by the constructivist and socio-constructivist learning theories, student-centred teaching methods have been introduced [23]. One of the most studied integrated curriculums based on socio-constructivist theory is the problem-based curriculum (PBL). PBL is a student-centred teaching method, in which students discuss cases in small groups, reactivating their previous knowledge and constructing coherent explanations of the problem, in order to prepare their self-study. After this period, students meet again and share how they understood and solved the case [24]. A major strength of PBL is that it promotes integration of knowledge and skills. Students not only acquire knowledge but also practice problem-solving skills, critical thinking, small group work and autonomous learning [25,26]. These constructivist learning environments are thought to influence positively students’ perception of their educational context and to foster deep learning [13,27].

Identifying the elements of the educational context enhancing students’ perception might give cues to improve their learning experience. However, the nature of the links between the educational context itself, the way in which it is perceived by students and students’ learning approaches is poorly understood. This study aimed at investigating in a multi-institutional natural environment the relationships between students’ perception of their educational context and learning approaches in three learning environments differing by their teaching formats (lecture or problem-based-learning PBL) and integration level of the curriculum (traditional or integrated). We used path analysis modelling to test the hypothesis that a PBL format and an integrated curriculum are associated to deeper approaches to learning and that this is mediated by student perception.

## Material and methods

### Participants

A total of 2734 first-to-third-year medical students were recruited during the academic years 2012–2013 to 2015–2016 in three different French-speaking medical schools (centre 1: Lyon, France, first study year, LY1; centre 2: Lausanne, Switzerland, first study year, LA1; centre 3: Geneva, Switzerland, first, second and third study years, GE1, GE2 and GE3, respectively). From the 2734 students, 1739 (64%) answered and returned the questionnaires. The return rate was about 29% in centre 1 due to the free on-line access and was between 88 and 98% in centres 2 and 3 in which students were contacted in class and answered paper questionnaires (Table 1). In centre 1, the respondents were representative of the whole group in terms of age, gender, proportion of repeaters and type of high school diploma.

**Table 1.** Description of the population, of the administration of questionnaires and of the educational context.

	Educational context^a^					
	A Traditional lecture-based	B Integrated lecture-based		C Integrated PBL-based		
*Population and administration*						
Class	LY1^2^	LA1^3^	GE1^4^	GE2^5^	GE3^6^	All
Study year	1	1	1	2	3	
Medical school institutions	Lyon	Lausanne	Geneva	Geneva	Geneva	
*Questionnaire administration*	Online	Paper	Paper	Paper	Paper	
Number of students recruited	1767	435	380	320	207	3109
Number of questionnaires returned (%)	505 (29.0)	408 (93.8)	333 (87.6)	290 (90.6)	203 (98.1)	1739 (55.9)
Number of questionnaires eligible for analysis (%)	295 (16.8)	362 (83.2)	250 (65.8)	284 (88.7)	203 (98.1)	1394 (44.8)
Gender (% female)	67.8	67.6	66.1	56.7	55.7	63.4
Mean age (SD)	19.0 (1.1)	20.8 (2.6)	20.5 (1.3)	22.0 (1.9)	22.9 (2.2)	20.9 (2.3)
*Educational context*						
Curriculum type	Thematic modules	Thematic modules	Thematic modules	Thematic modules	Thematic modules	
Major teaching formats	Traditional lectures (90%)	Integrated lectures (40%); self-study (50%)	Integrated lectures (50%) self-study (40%)	Integrated PBL^7^ incl. self-study (90%)	Integrated PBL^7^ incl. self-study (90%)	
	Tutorials in large groups (10%)	Practical skills (10%)	Practical skills (10%)	Clinical skills teaching (10%)	Clinical skills teaching (10%)	
Major assessment formats	MCQ^8^ + SAQ^9^	MCQ^8^ + SAQ^9^	MCQ^8^ + SAQ^9^	MCQ^8^ + oral + OSCE^a0^	MCQ^8^ + oral + OSCE^a0^	
Success rate on the end-of-year exam (%)	17	40	30	98	98	

^a^ see methods for details; ^2^Lyon year 1; ^3^Lausanne year 1; ^4^Geneva year 1; ^5^Geneva year 2; ^6^Geneva year 3; ^7^Problem-based learning; ^8^Multiple-choice questions; ^9^Short-answer questions; ^10^Objective structured clinical exam

### Description of the educational contexts

In France and the French part of Switzerland, the undergraduate curriculum is divided into a selection year (1st year of study), two preclinical years (2nd and 3rd years), two clinical years (4th and 5th years) and one elective year (6th year). A licensing exam at the end of the sixth year ends the undergraduate medical training.

The curricula at the various institutions and study years differed by their teaching formats (lecture or problem-based-learning PBL) and integration level of the curriculum (traditional or integrated), which naturally provided three different educational contexts, in which to study students’ perception of their educational context and learning approaches. We used the integration ladder framework [22] to characterize the level of integration of the curriculums. LY1was characterized by a traditional curriculum essentially based on lectures given to large classes, and based on a disciplinary approach (‘isolation to awareness’ [22]). LA1 and GE1 were characterized by a curriculum also based on lectures given to large classes, but differing from the previous situation by a teaching steered by multidisciplinary working groups in order to ensure relevance with regard to medical studies and to coordinate and integrate inside and between the modules (‘nesting’, ‘temporal coordination’, ‘sharing’, ‘correlation’ [22]). GE1 had in addition an Integration module (teaching integrative subjects such as Adaptation to effort and Inflammation, capitalizing on knowledge acquired during the previous modules) and linking cases (teaching cystic fibrosis and atherosclerosis to illustrate on-time all along the year the molecular, cellular, organ or systemic aspects taught in the modules: ‘complementary’ [22]). GE2 and GE3 were characterized by a curriculum constituted of multidisciplinary thematic teaching units taught by PBL. In each unit, students acquired knowledge from several disciplines integrated in each case, following the classical format of tutorial (case opening), self-directed study and reporting (case wrap-up and analysis of the learning process and of the group functioning) sessions (about 4 hrs tutorials and 24 hrs of self-learning per week). A few lectures and practical sessions (about 2 and 4 hrs per week, respectively), seminars of clinical skills training and community dimension (about 6 hrs per week) ran in parallel and were coordinated with problems (‘complementary’, ‘multi-disciplinary’ [22]).

Three educational contexts were defined according to the teaching format (lecture or PBL) and to the integration level of the curriculum (traditional or integrated). Thus, LY1 was defined as traditional lecture-based (educational context A), LA1 and GE1 as integrated lecture-based (educational context B), GE2 and GE3 as integrated PBL-based (educational context C) (Table 1).

### Data collection

#### Instruments

Data used in the study derived from students’ self-reported answers to 2 instruments (supplementary tables A and B).

*Learning approaches* were measured with the Revised two-factor Study Process Questionnaire (R-SPQ-2F), developed by Biggs et al. [4]. The original version consisted of 20 items scored from 1 (*this item is never or only rarely true for me*) to 5 (t*his item is always or almost always true for me*), with 2 major subscales: the Deep Approach (DA) and the Surface Approach (SA). The R-SPQ-2F being potentially culturally-sensitive [28,29], the French version presented by Gustin [30] was used after a minimal validation to ensure a reasonable application in our context. Exploratory factor analysis with two factors and geomin rotation led us to eliminate items 3 and 7 of SA. The confirmatory factor analysis model obtained with 18 items gave an acceptable fit (Root Mean Square Error of Approximation (RMSEA): 0.059; Comparative Fit Index (CFI): 0.916). Cronbach coefficients of the 18-items questionnaire were 0.78 and 0.71 for DA and new SA, respectively (see supplementary Table A for details).

*Students’ perception of the educational context* was measured with the Dundee Ready Educational context measure (DREEM), developed by Roff [31]. The original version consisted of 50 items scored from 0 (*strongly disagree*), to 4 (*strongly agree*) with 5 subscales. The perception of learning (12 items) related to the type of teaching (e.g., student-centred, stimulating, focused, emphasizing long-term learning, etc.). The perception of teachers (11 items) related to their attitudes, knowledge, preparation, ability to provide constructive feedback, etc. The academic self-perception (8 items) related to students’ own academic development (e.g., prepared for the profession, developing problem-solving skills, learning relevant for the future career, etc.). The perception of atmosphere (12 items) related to the general feeling of relaxed teaching activities, enjoyment outweighing stress, possibility of concentrating well, etc. The social self-perception (7 items) related to the support system for students, their social life, the interest of the matter, etc. The DREEM was considered as the most suitable instrument to measure students’ perceptions of their educational context in undergraduate medical education settings [32]. It had been used and validated in many languages and student populations, and was considered as a generic instrument to undergraduate health professions education and non-culturally sensitive [33]. The French version used for this study was a professional translation from the validated English version at the Ottawa University (courtesy of Timothy Willett). The confirmatory factor analysis model gave reasonable errors of approximation (RMSEA: 0.068; CFI: 0.81), and we decided to use it without any modification, because of the strong theoretical basis of the instrument. The respective Cronbach coefficients for the five subscales were 0.80, 0.69, 0.73, 0.78, 0.64 and 0.91 for the total scale (see supplementary Table B for details).

#### Administration of the survey

The survey was administered online in centre 1 and on paper in centres 2 and 3 during the second semester. In Lyon Faculty of Medicine, students received a letter from the Dean of medicine by e-mail a week before administering the questionnaires to inform them of the current study. With the agreement of the ethic comity of the *Hospices Civils* de *Lyon*, the questionnaires were proposed as free online access just after student took their mock exam. In Lausanne and Geneva Faculty of Medicine, students received an email 10 days before the survey to inform them about the research project’s main goals, the questionnaires’ content and the testing conditions (confidential, voluntary participation). All students present in the classroom on the survey day received this information once again. Students who agreed to participate signed a written consent form, as appropriate. The study was exempted from a formal review by the chair of the ethics committee of the State of Geneva (University representative).

### Statistical analysis

We considered eligible for the analyses data from students who completed both questionnaires with less than 5% of missing data by dimension. As such, from the returned questionnaires, 1394 were eligible for analyses. The mean scores of DA, SA, and of the five DREEM subscales were computed for each group. Pearson’s correlations were calculated between all observed variables. The differences in scores between the three educational contexts were computed with confidence intervals (95% CI) and effect sizes (negligible if <0.30 and practically important if >0.70 [34]).

#### Path analysis

Path analysis is a special case of structural equation modelling which is used to examine directed relationships between a set of observed variables. We tested 2 models by path analysis. Model 1 evaluated what in students’ perception of their educational context was related to their use of deep and surface learning approaches. It tested all the relationships from each DREEM subscale to deep and surface learning approaches and from student gender and age to each DREEM subscale and learning approaches. The model assumed that the 5 DREEM subscales were inter-correlated with one another as well as DA with SA. This model 1 was tested in all participants (Model1_All) and in each of the 5 classes in turn (Model1_Clas).

Model 2 evaluated the relationships from the three educational contexts to students’ use of deep and surface learning approaches. It tested the direct and indirect (i.e., mediated via students’ perception of their educational context) relationships. The model assumed that the 5 DREEM subscales were inter-correlated with one another as well as DA with SA. This model 2 was fitted in all participants (Model2_All) and 5 times after removing each class from the whole sample in turn (sensitivity analysis, Model2_WOClas) to evaluate the robustness of the model. Only standardized path coefficients were reported.

Descriptive analyses were performed in R language (version 3.3.0) available at https://cran.r-project.org/. Factorial and path analyses (structural equation modelling) were performed with Mplus (version 7.11) available at https://www.statmodel.com/. The root mean square error of approximation (RMSEA) and Comparative Fit Index (CFI) were used to appreciate model fit in path analysis [35,36]. A RMSEA value of ≤ 0.06 and a CFI ≥ 0.90 were considered as an acceptable fit.

## Results

### Descriptive statistics

Students’ perceptions of their educational context as well as their learning approaches were statistically different in the three educational contexts on all dimensions (Table 2). It was better in the PBL-based integrated curriculum (C: total 136.3 ± 17.3), followed by the lecture-based integrated curriculum (B: 123.3 ± 19.8) and finally by the lecture-based traditional curriculum (A: 105.4 ± 21.3). Students used equivalent deeper and less surface approaches in both the PBL-based and lecture-based integrated curricula (33.7 ± 5.7 and 33.7 ± 6.1; 23.7 ± 5.9 and 24.7 ± 6.5 for DA and SA, respectively). In the traditional lecture-based curriculum, however, students used about as much deep (29.5 ± 5.9) than surface (27.6 ± 7.1) approaches to learning.

**Table 2.** Descriptives of students’ perception of their educational context and of learning approaches: mean scores (SD) and change in scores of variables by educational context (95% confidence interval CI and effect sizes).

	Educational context^a^									
	A	B			C			change in scores]95%CI[(effect sizes)		
Class (N)	LY1^2^ (295)	LA1^3^ (362)	GE1^4^ (250)	all	GE2^5^ (284)	GE3^6^ (203)	all	A to B	A to C	B to C
**Students’ perception of their educational context**										
Total	105.4 (21.3)	*122.0* *(20.8)*	*125.2* *(18.2)*	123.3 (19.8)	*139.5* *(16.3)*	*131.7* *(17.7)*	136.3 (17.3)	17.9]14.7;21.1[(0.88)	30.8]27.5;34.2[(1.63)	12.9 ]10.2;15.7[ (0.69)
Learning	23.3 (6.4)	*28.5* *(6.3)*	*29.3* *(5.6)*	28.8 (6.0)	*31.7* *(5.0)*	*30.2* *(5.4)*	31.0 (5.2)	5.5 ]4.5;6.5[ (0.89)	7.7 ]6.7;8.7[(1.36)	2.2 ]1.4;3.1[ (0.40)
Teaching	24.7 (5.2)	*28.0* *(4.4)*	*30.1* *(4.7)*	28.9 (4.7)	*31.2* *(3.9)*	*28.7* *(4.1)*	30.1 (4.2)	4.2 ]3.4;4.9[ (0.86)	5.4 ]4.6;6.2[(1.18)	1.3 ]0.6;1.9[ (0.28)
Academic	15.2 (5.0)	*17.0* *(5.0)*	*18.1* *(4.2)*	17.4 (4.8)	*21.8* *(3.8)*	*20.6* *(3.8)*	21.3 (3.9)	2.2 ]1.5;3[ (0.46)	6.1 ]5.3;6.8[(1.40)	3.8 ]3.2;4.5[ (0.88)
Atmosphere	27.2 (6.3)	*30.3* *(6.5)*	*30.0* *(5.8)*	30.2 (6.2)	*35.5* *(4.6)*	*33.8* *(5.3)*	34.8 (5)	3 ]2.1;4[ (0.49)	7.6 ]6.6;8.7[(1.39)	4.6 ]3.8;5.4[ (0.81)
Social	15.1 (4.3)	*18.3* *(3.9)*	*17.7* *(3.5)*	18.0 (3.7)	*19.4* *(3.4)*	*18.5* *(3.4)*	19.0 (3.4)	3 ]2.4;3.6[ (0.76)	4 ]3.3;4.6[(1.04)	1 ]0.5;1.5[ (0.27)
**Students’ learning approaches**										
Deep	29.5 (5.9)	*33.9* *(6.0)*	*33.3* *(6.1)*	33.7 (6.1)	*34.3* *(5.3)*	*32.8* *(6.17)*	33.7 (5.7)	4.2 ]3.2;5.2[ (0.69)	4.2 ]3.1;5.2[(0.72)	0 ]-0.9;0.8[ (0.00)
Surface	27.6 (7.1)	*24.6* *(6.4)*	*24.9* *(6.6)*	24.7 (6.5)	*22.9* *(5.6)*	*24.8* *(6.1)*	23.7 (5.9)	−2.8 ]-3.9;-1.8[ (−0.43)	−3.9 ]-5;-2.8[ (−0.61)	−1.1 ]-2;-0.1[ (−0.17)

^a^ see methods for details; ^2^Lyon year 1; ^3^Lausanne year 1; ^4^Geneva year 1; ^5^Geneva year 2; ^6^Geneva year 3

Correlations between variables were low to moderate (Table 3).

**Table 3.** Correlations between variables in each educational context.

	A (N 295)							B (N 612)							C (N 487)						
Educational context	1	2	3	4	5	6	7	1	2	3	4	5	6	7	1	2	3	4	5	6	7
1. deep approach to learning	_							_							_						
2. surface approach to learning	−0.15	_						−0.34	_						−0.34	_					
3. student perception of learning	0.36	−0.28	_					0.42	−0.28	_					0.30	−0.20	_				
4. student perception of teacher	0.11	−0.18	0.55	_				0.18	−0.15	0.56	_				0.17	−0.14	0.58	_			
5. student academic self-perception	0.38	−0.26	0.67	0.37	_			0.38	−0.17	0.63	0.40	_			0.34	−0.13	0.61	0.41	_		
6. student perception of atmosphere	0.27	−0.22	0.57	0.50	0.53	_		0.26	−0.19	0.63	0.43	0.48	_		0.27	−0.19	0.67	0.51	0.59	_	
7. student social self-perception	0.15	−0.31	0.42	0.31	0.43	0.64	_	0.25	−0.16	0.52	0.30	0.38	0.61	_	0.17	−0.11*	0.54	0.33	0.49	0.64	_

All correlations are significant at the *p* < 0.001 level except * (*p* < 0.05)

### Relationships between students’ perception of their educational context and their learning approaches

**Figure 1.**
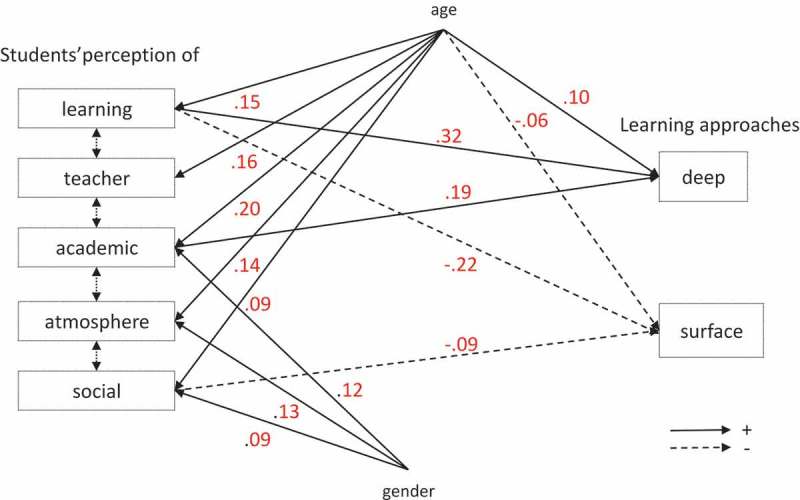
Path analysis model showing the relationships from students’ perception of their educational context on their learning approaches (only significant relationships are indicated). Model 1 tested all the relationships from each DREEM subscale on deep (DA) and surface (SA) learning approaches and from gender on each DREEM subscale and on deep and surface learning approaches. The model assumed that the 5 DREEM subscales were inter-correlated with one another as well as DA with SA. Only significant relationships with their beta coefficients are represented on the figure. Full lines are positive relationships and dotted lines negative relationships.

Model 1_All perfectly fitted our data (RMSEA = 0; CFI = 1; Figure 1 and Table 4). Students’ use of deep learning approaches was increased by their perception of good learning and academic self-perception (e.g., students increased their use of deep approaches of 0.32 SD for an increase of 1 SD in their perception of learning). Students’ use of surface approaches was decreased by their perception of good learning and marginally by their social self-perception. These effects were absent in some classes: for example, students’ perception of learning was not associated with their use of learning approaches in GE3 for DA and SA and in LY1 for SA. On the contrary, students’ social self-perception was associated to SA only in LY1. Older students used more DA and less SA, and perceived better their educational context on all dimensions. Male students had a better perception of the atmosphere, as well as of their academic and social self-perceptions. The model explained 22% and 12% of the variances of DA and SA respectively.

**Table 4.** Standardized coefficients of the paths from each dimension of students’ perception of their educational context on their learning approaches (all models 1).

			ANALYSIS BY CLASS				
			models 1.Clas				
Dependent	Explicative						
Variables	Variables	ALL model 1.All	LY1	LA1	GE1	GE2	GE3
**Deep approach**	Learning	0.32***	0.24**	0.19**	0.50***	0.26**	0.09
	Teacher	−0.06	−0.14	−0.07	−0.06	−0.05	−0.08
	Academic	0.19***	0.23**	0.31***	0.11	0.20**	0.28**
	Atmosphere	−0.02	0.13	−0.06	−0.05	0.07	0.12
	Social	0.03	−0.09	0.14*	−0.06	−0.01	−0.14
	Male vs. Female	−0.03	0.01	−0.10*	0.09	−0.01	−0.14*
	Age	0.10***	0.01	0.15*	0.09	0.12*	0.11
**Surface approach**	Learning	−0.22***	−0.14	−0.31***	−0.20*	−0.30***	0.02
	Teacher	−0.03	−0.02	0.03	−0.06	0.01	0.03
	Academic	−0.02	−0.11	0.04	−0.05	0.06	−0.03
	Atmosphere	−0.02	0.06	0.01	−0.07	−0.04	−0.20
	Social	−0.09*	−0.25**	−0.02	−0.03	0.02	0.06
	Male vs. Female	0.03	0.12*	0.06	−0.11	0.05	−0.01
	Age	−0.06*	0.02	−0.05	−0.07	−0.05	−0.07
Learning	Male vs. Female	0.05	0.13*	−0.01	0.05	−0.06	0.04
	Age	0.15***	−0.01	−0.08	−0.11	−0.18*	−0.10
Teacher	Male vs. Female	0.03	0.07	−0.12*	0.05	0.04	0.05
	Age	0.16***	−0.02	−0.01	−0.05	0.01	−0.09
Academic	Male vs. Female	0.12***	0.19**	0.06	0.09	0.07	0.08
	Age	0.20***	0.03	−0.07	0.01	−0.08	−0.03
Atmosphere	Male vs. Female	0.13***	0.25***	0.07	0.11	0.06	0.05
	Age	0.14***	−0.10*	−0.14	−0.08	−0.11*	−0.12
Social	Male vs. Female	0.09**	0.11	0.07	0.14*	0.03	0.03
	Age	0.09**	−0.14*	−0.10	−0.13	−0.13**	−0.17**

Significant standardized coefficients are in bold; *** *p* ≤ 0.001; ** 0.001 < *p* ≤ 0.01; * 0.01 < *p* ≤ 0.05 Model 1.All was fitted in all pooled classes and models 1.Clas separately in each class

### Direct and mediated relationships from each educational context to students’ learning approaches

**Figure 2.**
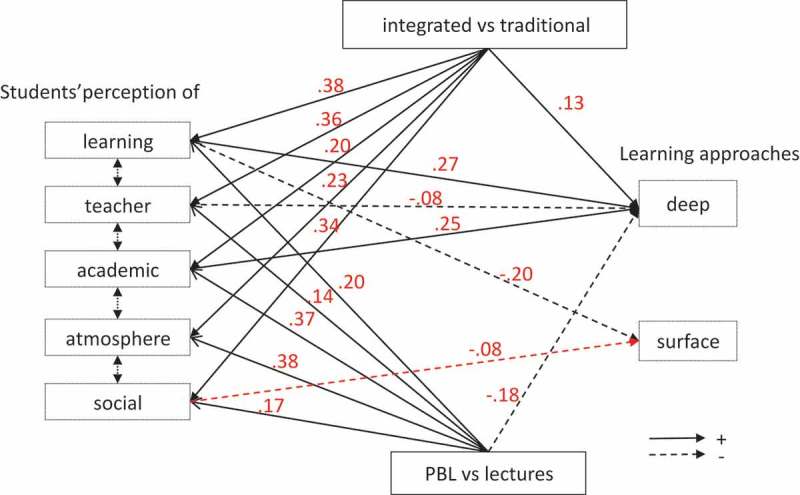
Path analysis model showing the relationships from the integration level and the teaching format of the curriculum on students’ perception of their educational context and on their learning approaches (only significant relationships are indicated). Model 2 tested the relationships from the integration level and the teaching format of the curriculum on students’ use of deep (DA) and surface (SA) learning approaches, postulating a direct path and an indirect path mediated via students’ perception of their educational context. The model assumed that the 5 DREEM subscales were inter-correlated with one another as well as DA with SA. Only significant relationships between integration level, teaching format, students’ perception and students’ learning approaches are represented with their beta coefficients on the figure. Full lines are positive relationships and dotted lines negative relationships.NB: The relationships between gender, age and the other variables are not represented for a matter of clarity but can be found in Table 5. All coefficients are adjusted for gender and age.

Model 2_All perfectly fitted our data (RMSEA = 0; CFI = 1; Figure 2 and Table 5) hence supporting the hypothesis that students’ learning approaches were related to the educational context. In addition it showed that this relationship was constituted of 2 paths, one direct from the educational context to the learning approaches, and one indirect mediated by students’ perception of this educational context.

**Table 5.** Standardized coefficients of the paths from the integration level of the educational context and from each dimension of students’ perception of these educational contexts on their learning approach (all models 2).

			WITHOUT (SENSITIVITY ANALYSIS)				
			(models 2_WoClas)				
DependentVariables	ExplicativeVariables	ALL(model 2_All)	LY1	LA1	GE1	GE2	GE3
**Deep**	Learning	0.27***	0.27***	0.29***	0.21***	0.27***	0.31***
**Approach**	Teacher	−0.08*	−0.06	−0.09*	−0.07	−0.10*	−0.09*
	Academic	0.25***	0.25***	0.23***	0.29***	0.23***	0.23***
	Atmosphere	0.05	0.02	0.10	0.05	0.05	0.03
	Social	−0.01	0.01	−0.07	0	−0.01	0.01
	Male vs. Female	−0.03	−0.04	−0.01	−0.06	−0.04	−0.01
	Age	0.11***	0.12***	0.09**	0.11***	0.12***	0.11***
	Integrated vs. Traditional	0.13***		0.12**	0.18***	0.14***	0.13***
	PBL vs. lectures	−0.18***	−0.20***	−0.15***	−0.21**	−0.19***	−0.12***
**Surface**	Learning	−0.20***	−0.22***	−0.17**	−0.20***	−0.18***	−0.24***
**Approach**	Teacher	−0.02	−0.02	−0.04	−0.01	−0.01	−0.02
	Academic	−0.02	0.01	−0.05	−0.02	−0.04	−0.01
	Atmosphere	−0.03	−0.06	−0.04	−0.02	−0.02	0
	Social	−0.08*	0	−0.09*	−0.09*	−0.09*	−0.10*
	Male vs. female	0.03	0	0.03	0.06*	0.03	0.04
	Age	−0.04	−0.05	−0.03	−0.04	−0.05	−0.04
	Integrated vs. traditional	−0.06		−0.06	−0.07	−0.06	−0.05
	PBL vs. lectures	0	0	0.01	−0.01	0.06	−0.04
Learning	Male vs. female	0.03	0	0.04	0.02	0.05	0.03
	Age	−0.11**	−0.13**	−0.13***	−0.11**	−0.08	−0.10*
	Integrated vs. traditional	0.38***		0.45***	0.38***	0.40***	0.39***
	PBL vs. lectures	0.20***	0.24***	0.19***	0.22***	0.11**	0.21***
Teacher	Male vs. female	0.01	0	0.04	0	0.01	0.01
	Age	−0.06*	−0.07*	−0.08	−0.05	−0.04	−0.02
	Integrated vs. traditional	0.36***		0.49***	0.31***	0.38***	0.36***
	PBL vs. lectures	0.14***	−0.16***	0.03	0.22***	0	0.19***
Academic	Male vs. female	0.09***	0.07*	0.10***	0.09**	0.10***	0.09***
	Age	−0.06	−0.07*	−0.04	−0.06	−0.04	−0.05
	Integrated vs. traditional	0.20***		0.27***	0.17***	0.21***	0.20***
	PBL vs. lectures	0.37***	0.42***	0.32***	0.42***	0.25***	0.36***
Atmosphere	Male vs. female	0.10***	0.07*	0.11**	0.10***	0.12***	0.11***
	Age	−0.13**	−0.13**	−0.13***	−0.14**	−0.13**	−0.11**
	Integrated vs. traditional	0.23***		0.24***	0.25***	0.25**	0.24***
	PBL vs. lectures	0.38***	0.42***	0.42**	0.37***	0.26***	0.37***
Social	Male vs. female	0.07**	0.07*	0.07*	0.06*	0.08**	0.08**
	Age	−0.14***	−0.14***	−0.18***	−0.14***	−0.13***	−0.12***
	Integrated vs. traditional	0.34***		0.34***	0.34***	0.36***	0.35***
	PBL vs. lectures	0.17***	0.19***	0.24***	0.09**	0.09**	0.18***

Significant standardized coefficients are in bold; *** *p* ≤ 0.001; ** 0.001 < *p* ≤ 0.01; * 0.01 < *p* ≤ 0.05 Model 2. All was fitted in all pooled classes and models 2.WoClas after removing each class from the whole sample. Grey areas indicate that the effect of partial vs. no integration could not be estimated since the only class with no-integration (i.e., LY1) was removed from the whole sample

*Direct path*: In the lecture-based curricula, integration increased the use of deep approaches, whereas in PBL compared to lectures (in an integrated curriculum) the use of deep approaches decreased. Both had no effect on surface approaches. As in Model 1, older students used deeper approaches. The sensitivity analysis (model 2_WoClas) confirmed these findings.

*Indirect path*: Integration (integrated compared to traditional curriculum) and PBL (compared to lectures) improved students’ perception of all dimensions. Integration had more effect on students’ perception of learning, of teacher and of social self-perception, whereas PBL particularly improved students’ academic and atmosphere perceptions. This improved students’ perception (learning and academic) influenced in turn students’ use of deep and surface approaches (like in Model 1). In each of the three educational contexts, older students were less positive in their perceptions than younger students (except for academic perception), and this in turn also influenced their use of deep and surface approaches.

The model explained 25% and 12% of the variances of DA and SA, respectively.

Table 6 summarized the direct and indirect paths from the educational context to the deep and surface learning approaches. Compared to a traditional lecture-based curriculum, an integrated lecture-based curriculum increased deep (0.26) and decreased surface (−0.18) approaches, cumulating the effects of the direct and indirect paths. The PBL-based integrated curriculum had no additional effect (−0.03) on students’ use of deep learning approaches, compared to an integrated lecture-based curriculum: it combined two opposite effects, a direct negative (−0.18) and an indirect positive (0.15) effects. In addition, it had a small negative effect (−0.08) on students’ use of surface approaches, through the indirect path. Age, also combining opposite direct and indirect effects, had finally no effect on students’ use of surface approaches and a small positive effect (0.07) on students’ use of deep approaches. Gender had no significant effect on these relationships.

**Table 6.** Summary of the direct and indirect paths from the integration level of the curriculum, from the teaching format and from gender to the deep and surface learning approaches.

	Effect	On deep approach	On surface approach
integrated vs. traditional (lecture-based)	Direct	**0.13** (0.11;0.18)	−0.06§ (−0.10;-0.05)
	Indirect	**0.13** (0.09;0.15)	**−0.12** (−0.15;-0.09)
	Total	**0.26** (0.20;0.29)	**−0.18** (−0.21;-0.19)
PBL vs. Lectures (integrated)	Direct	**−0.18** (−0.21;0.03)	0§ (−0.04;0.06)
	Indirect	**0.15** (0.09;0.17)	**−0.08** (−0.09;-0.04)
	Total	−0.03§ (−0.09;0.11)	**−0.08** (−0.12;0.01)
Male vs. Female	Direct	−0.03§ (−0.06;0)	0.033§ (0;0.06)
	Indirect	**0.03** (0.02–0.04)	−0.016§ (−0.03;0)
	Total	0§ (−0.02;0.03)	0.017§ (0;0.05)
Age	Direct	**0.11** (0;0.12)	−0.04§ (−0.05;0)
	Indirect	**−0.04** (−0.05;0.03)	**0.04** (0.03;0.05)
	Total	**0.07** (0.03;0.09)	0§ (−0.02;0.03)

Standardized coefficients (range of the sensitivity analysis); § not significant at 5% level

## Discussion

This study aimed at investigating by path analysis the relationships between students’ perception of their educational context and their learning approaches in three learning environments differing by their teaching formats (lecture or PBL) and integration level of the curriculum (traditional or integrated). We found in this specific setting that an integrated lecture-based curriculum seemed as effective in stimulating students’ use of deep learning approaches as a PBL-based curriculum. In addition, the analysis suggested that the educational context is related to learning approaches by two paths, one direct and one mediated by student perception, providing some new insights into the complex interplay between the educational context and students’ learning approaches. Indeed our analysis suggested that both paths had cumulative effects in the case of the integrated lecture-based curriculum (compared to the traditional), whereas they had opposite effects in the case of the PBL-based curriculum (compared to the integrated lecture based).

Findings of this study show that, compared to a traditional curriculum, an integrated curriculum, even if based on lecturing, improved students’ perception of their educational context on all dimensions (learning, teacher, general atmosphere, academic and social self-perceptions). Furthermore, when taught by PBL it increased even more the positive way students perceived their educational context. This is different from a previous study in which lecture-taught students’ perceptions were positive, but perception of activating methods was very variable with extremely positive and negative opinions [37]. An analysis of the differences between the three curricula highlighted the following points. In the lecture-based traditional curriculum, teaching was not coordinated, teaching methods were focused on transmission and promoted rote learning, and a severe selection pressured students and induced a climate of fear of failure. In the lecture-based integrated curriculum, modules were constructed by multidisciplinary teams, learning objectives were coordinated around different disciplines, red threads were emphasized inside and throughout the modules (e.g., by the linking cases), and lectures were contextualized. We pondered that this context, although similarly competitive, allowed students to give meaning to what was taught, to form relationships among concepts and to connect their learning to future needs [13,21,22]. In the PBL-based curriculum, perception was even better because of the teaching organized around realistic clinical cases and students being active and cooperative learners. This could improve their perception of learning, of feeling confident in their academic success, and of being prepared to the exercise of their profession [13,25,27,38]. This could also enhance their social self-perception through repeated practice of group work [39].

Our path analysis models were consistent with published research, showing that students tended to adopt one approach or the other depending on the context in which they were learning [4,5,10,15] and that not only the context itself but also how it is perceived by students was influencing their approaches to learning [13,14]. With regards to the model proposed by J. Biggs [5], we found that the way students perceived their teachers seemed to have little or no influence on their learning approaches. By contrast, their perception of the type of teaching (e.g., student-centred, stimulating, focused, emphasizing long-term learning) as well as of their own academic development (e.g., prepared for the profession, developing problem-solving skills, learning relevant for the future career) did impact the way they learned, stimulating the use of deep and decreasing the use of surface approaches.

Like previous studies, we observed that a traditional not integrated subject-based curriculum increased surface approaches [40,41]. In addition, the conditions of strong selection encountered by first-year students could be experienced as a ‘survival course’ and induce students to adopt surface strategies suited to the learning situation [42]. Interestingly, however, we noted that in similarly competitive contexts, the Swiss first-year students perceived their context as better and adopted less surface approaches than the French first-year students. We hypothesize that a potentially different social climate among the three institutions, aided by an integrated curriculum including clear objectives, defined assessment criteria and relevant teaching content, allowed to optimize and align the curriculum, thus eventually favouring deep learning [43–45]. When it came to compare the lecture-based to the PBL-based integrated curricula, the model suggested that the PBL format on one hand improved students’ perception of their educational context which in turn stimulated them to use deeper and less surface learning approaches, but on the other hand directly decreased students’ use of deep approaches, with no effect on surface approaches. The finding that PBL might have two opposite effects on learning approaches could provide cues to understand some of the controversial findings about the effect of PBL on learning approaches. Indeed in a recent review, Dolmans and colleagues [46] found that PBL had an overall small positive effect on the deep approach and no effect on the surface approach. However, looking at individual studies effect sizes, it was striking to see that they ranged from −0.53 to 0.93 for the deep approach, and from −0.50 to 0.50 for the surface approach. The density of the curriculum, unclear goals, perception of cognitive load, insufficiently aligned assessment and workload were some of the factors that have been shown to counter-influence the desired outcome of PBL and other constructivist learning environments on stimulation of deep approach [12,44,47–49]. In this line, a previous study suggested that the practice of PBL may evolve along a PBL-based curriculum and move away from the original model [50]. A previous qualitative analysis of our students’ comments revealed that the perceived workload, the insufficiently aligned assessment and the absence of a counterbalanced role of teachers were the factors driving the learners away from the deep learning planned with PBL (unpublished results). In addition, the complexity and time-consuming learning activities required in PBL sessions [48] and the demands of the deep learning approach over the surface approach could induce students to revert over the years to a more expedient, superficial and hence less demanding approach to learning [9,11,12].

The present study highlighted the complexity and difficulty to influence students’ approaches towards deep learning [13]. Our models suggested that about 25% of the variance of students’ deep approaches might be explained by the impact of their educational context and their perception. The other factors belong to the personal and institutional fields. The institutional culture can influence both the educational context itself and its perception by students [51]. However, the impact of institutional factors is about four times smaller than that of individual factors on the perceptions of the educational context [52]. Yet, perceptions being defined through the learner’s lens, this makes the educational context fairly complex to be defined and understood [52]. One of the most influencing factors seems to be students’ initial learning approach [53,54], that could be driven by their epistemological beliefs [55]. It impacts how students approach learning, whatever the characteristics of the course are, and seem to account for about 30% of the variance in students’ subsequent use of learning approaches [12]. Moreover, the stronger the initial learning approaches, the less they may subsequently change according to the educational contexts (Gijbels 2008), since they influence the appreciation and use of elements of the educational context, reducing the impact of instructional measures [54]. For example, deep learners preferring educational contexts favouring understanding were more positive in their perception, seemed more suited to PBL than surface learners [56] and did not change their approach between a conventional and a PBL curriculum [12,54]. Conversely, surface learners adapted their learning approaches in action learning (interactive lectures and group work) and utilized deeper strategies than in a conventional curriculum [54]. This suggests that future studies should include personal characteristics of students, in particular their initial learning profile that might influence their individual evolution of learning approaches during their study years.

Strengths of this study are first its multi-institutional and international design. Having similar findings at three medical schools from two countries and three different study years, could provide the representativeness to support the generalizability of the findings concerning the impact of students’ perception of the educational context on learning approaches. Second, the use of path analysis to test and confirm our hypotheses allowed for the disentangling between direct and mediated effects of the educational context on learning approaches.

Limitations of this study concern first the use of translated scales for DREEM and R-SPQ-2F, which could impact some psychometric properties of the instruments. Second, the use of self-administered questionnaires which have been claimed inaccurate and potentially limiting the interpretation of the results; yet, self-perception may still influence how students learn. Third, the lower return rate from the class LY1 could limit the representability of the lecture-based not integrated group. However we have showed evidence that its socio-demography was representative of the whole population, and have no reason to think that the learning approaches adopted by these students would be different. In addition, the sensitivity analysis ensured that it did not much influence the results. A fourth limitation concerns all the factors that might influence learning approaches and were not considered in this study, such as student and teacher factors [13], and institution-specific influences [57].

In conclusion, our study suggests that a carefully integrated lecture-based curriculum was as effective as a PBL-based curriculum in stimulating students to adopt deep approaches to learning. This does not mean that PBL must be challenged, since there is sufficient evidence to prove that PBL is a very effective teaching format [23,58,59]. However, the way PBL is practiced should be questioned, since its effectiveness highly depends on its implementation [60]. Thus, curriculum design could benefit of putting its forces first in multidisciplinary teams developing a student-centred, integrated, multidisciplinary program, before choosing the teaching format itself, in order to create favourable conditions for deep learning [61].
