# Supplementary material for: Integrated problem-based learning versus lectures: a path analysis modelling of the relationships between educational context and learning approaches
Source: Med Educ Online. 2018 Jul 3;23(1):1489690. doi: 10.1080/10872981.2018.1489690 (PMC6041782; doi:10.1080/10872981.2018.1489690)

Supplementary data

Table A: Standardized coefficients of the final confirmatory factor analysis structure for the 18-items French version of the R-SPQ-2F in the total sample (n = 1394)

|  |  | Standardized coeficients | |
| --- | --- | --- | --- |
| **item** |  | **DA** | **SA** |
| 1 | Studying a topic gives me at times a feeling of satisfaction and accomplishment | 0.53 | - |
| 2 | I am only satisfied when I have studied a topic so much that I understand it deeply | 0.50 | - |
| 5 | I feel that almost every topic is interesting once I get into it deeply | 0.47 | - |
| 6 | I often spend extra time on new interesting topics | 0.73 | - |
| 9 | I find studying topics for my study at times as interesting as watching a movie or reading a novel | 0.60 | - |
| 10 | I test myself on important topics until I understand them completely | 0.46 | - |
| 13 | I work hard at my study because I find my study interesting | 0.63 | - |
| 14 | I spend a lot of my free time to know more about interesting topics dealt with in my courses | 0.70 | - |
| 17 | I come to most classes with questions in mind that I want answering | 0.37 | - |
| 18 | I take care that I did study various resources before the discussion in the groups | 0.41 | - |
| 4 | I only study seriously those topics that are mentioned in the course book or given by the tutor | - | 0.65 |
| 8 | I learn some things by rote, going over and over them until I know them by heart even if I do not understand them | - | 0.26 |
| 11 | I find I can get by in most assessments by memorizing key sections rather than trying to understand them | - | 0.32 |
| 12 | I generally restrict my self-study to what is specifically set as I think it is unnecessary to do anything extra | - | 0.77 |
| 15 | I find it not very meaningful to study topics in depth; it confuses and wastes times, when all you need is superficial knowledge of the topics | - | 0.54 |
| 16 | I believe that tutors shouldn’t expect students to spend much time on topics that will not be examined | - | 0.47 |
| 19 | I see no importance in studying topics which are not likely to be examined | - | 0.55 |
| 20 | The best way to pass examinations is in my opinion to try to remember as much answers to likely questions | - | 0.28 |

DA= deep approach ; SA= surface approach ; all coefficients are significant at α<0.001; items 3 and 7 were eliminated (see text); RMSEA=0.059, CFI=0.927

Table B. Standardized coefficients of the confirmatory factor analysis structure for the 50-items and 5-factor French version of the DREEM in the total sample (n = 1394)

|  |  | Standardized coefficients | | | | |
| --- | --- | --- | --- | --- | --- | --- |
| item |  | **learning** | **teacher** | **academic** | **atmosphere** | **social** |
| 1 | I am encouraged to participate during teaching sessions | 0.51 |  |  |  |  |
| 7 | The teaching is often stimulating | 0.58 |  |  |  |  |
| 13 | The teaching is student-centred | 0.63 |  |  |  |  |
| 16 | The teaching helps to develop my competence | 0.60 |  |  |  |  |
| 20 | The teaching is well-focused | 0.73 |  |  |  |  |
| 22 | The teaching helps to develop my confidence | 0.70 |  |  |  |  |
| 24 | The teaching time is put to good use | 0.46 |  |  |  |  |
| 25 | The teaching over-emphasizes factual learning | 0.33 |  |  |  |  |
| 38 | I’m clear about the learning objectives of the course | 0.48 |  |  |  |  |
| 44 | The teaching encourages me to be an active learner | 0.69 |  |  |  |  |
| 47 | Long-term learning is emphasized over short-term learning | 0.49 |  |  |  |  |
| 48 | The teaching is too teacher-centered | 0.33 |  |  |  |  |
| 2 | The teachers are knowledgeable |  | 0.36 |  |  |  |
| 6 | The teachers are patient with students |  | 0.57 |  |  |  |
| 8 | The teachers ridicule the students |  | 0.49 |  |  |  |
| 9 | The teachers are authoritarian |  | 0.30 |  |  |  |
| 18 | The teachers have good communication skills with students |  | 0.66 |  |  |  |
| 29 | The teachers are good at giving feedback to students |  | 0.62 |  |  |  |
| 32 | The teachers provide constructive criticism here |  | 0.72 |  |  |  |
| 37 | The teachers give clear examples |  | 0.60 |  |  |  |
| 39 | The teachers get angry in teaching sessions |  | 0.06 |  |  |  |
| 40 | The teachers are well-prepared for their teaching sessions |  | 0.48 |  |  |  |
| 50 | The students irritate the teachers* |  | 0.53 |  |  |  |
| 5 | Learning strategies which worked for me before continue to work for me now |  |  | 0.35 |  |  |
| 10 | I am confident about my passing this year |  |  | 0.51 |  |  |
| 21 | I feel I am being well prepared for my profession |  |  | 0.79 |  |  |
| 26 | Last year's work has been a good preparation for this year's work |  |  | 0.46 |  |  |
| 27 | I am able to memorize all I need |  |  | 0.37 |  |  |
| 31 | I have learned a lot about empathy in my profession |  |  | 0.51 |  |  |
| 41 | My problem-solving skills are being well developed here |  |  | 0.67 |  |  |
| 45 | Much of what I have to learn seems relevant to a career in healthcare |  |  | 0.62 |  |  |
| 11 | The atmosphere is relaxed during teaching units |  |  |  | 0.53 |  |
| 12 | The course is well time-tabled |  |  |  | 0.28 |  |
| 17 | Cheating is a problem in this course |  |  |  | 0.29 |  |
| 23 | The atmosphere is relaxed during lectures |  |  |  | 0.57 |  |
| 30 | There are opportunities for me to develop my interpersonal skills |  |  |  | 0.71 |  |
| 33 | I feel comfortable in class socially |  |  |  | 0.70 |  |
| 34 | The atmosphere is relaxed during seminars/tutorials |  |  |  | 0.65 |  |
| 35 | I find the experience disappointing* |  |  |  | 0.66 |  |
| 36 | I am able to concentrate well |  |  |  | 0.35 |  |
| 42 | The enjoyment outweighs the stress of the course |  |  |  | 0.62 |  |
| 43 | The atmosphere motivates me as a learner |  |  |  | 0.68 |  |
| 49 | I feel able to ask the questions I want |  |  |  | 0.56 |  |
| 3 | There is a good support system for students who get stressed |  |  |  |  | 0.43 |
| 4 | I am too tired to enjoy this course |  |  |  |  | 0.47 |
| 14 | I am rarely bored in this course |  |  |  |  | 0.60 |
| 15 | I have good friends in this course |  |  |  |  | 0.48 |
| 19 | My social life is good |  |  |  |  | 0.62 |
| 28 | I seldom feel lonely |  |  |  |  | 0.56 |
| 46 | My accomodation is pleasant |  |  |  |  | 0.25 |

All coefficients are significant at α<0.001; RMSEA=0.068; CFI=0.81


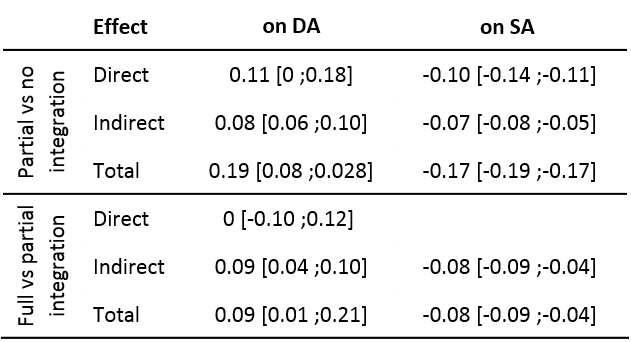

Supplement: Supplemental Material [file ZMEO_A_1489690_SM9893.docx]
